# Plants and Their Bioactive Constituents in Mesenchymal Stem Cell-Based Periodontal Regeneration: A Novel Prospective

**DOI:** 10.1155/2018/7571363

**Published:** 2018-08-05

**Authors:** Wenqing Xue, Jinhua Yu, Wu Chen

**Affiliations:** ^1^Key Laboratory of Oral Diseases of Jiangsu Province and Stomatological Institute of Nanjing Medical University, 140 Hanzhong Road, Nanjing, Jiangsu 210029, China; ^2^Department of Periodontics, School of Stomatology, Nanjing Medical University, 136 Hanzhong Road, Nanjing, Jiangsu 210029, China; ^3^Department of Endodontics, School of Stomatology, Nanjing Medical University, 136 Hanzhong Road, Nanjing, Jiangsu 210029, China

## Abstract

Periodontitis is a common chronic inflammatory disease, which causes the destruction of both the soft and mineralized tissues. However, current treatments such as bone graft materials, barrier membranes, and protein products all have difficulties in regenerating the complete periodontal tissue structure. Stem cell-based tissue engineering has now emerged as one of the most effective treatments for the patients suffering from periodontal diseases. Plants not only can be substrates for life processes, but also contain hormones or functional molecules. Numbers of preclinical studies have revealed that products from plant can be successfully applied in modulating proliferation and differentiation of human mesenchymal stem cells. Plant-derived substances can induce stem cells osteogenic differentiation, and they also possess angiogenic potency. Furthermore, in the field of tissue engineering, plant-derived compounds or plant extracts can be incorporated with biomaterials or utilized as biomaterials for cell transplantation. So it is speculated that botanical products may become a new perspective in stem cell-based periodontal regeneration. However, the lack of achieving predict clinical efficacy and quality control has been the major impediment to its extensive application. This review gives an overview of the prospect of applying different plant-derived substances in various human mesenchymal stem cells-based periodontal regeneration.

## 1. Background

Periodontitis is a set of chronic inflammatory disease which affects the periodontium. It can cause the irreversible destruction of the tooth-supporting tissues including alveolar bone, periodontal ligament (PDL), and root cementum. Periodontal regeneration is especially challenging, as it requires predictable regeneration of three quite diverse and unique tissues (e.g., cementum, PDL, and bone) and a triphasic interface between these different tissues to guarantee the restoration of their complex structure [1]. For decades, scientists have been seeking ways to repair the damage which occurs during periodontitis. They include the use of a range of surgical procedures, the use of a variety of grafting materials and growth factors, and the use of barrier membranes. But all these current treatment procedures just offer a limited potential for attaining complete periodontal restoration [2].

Stem cells are undifferentiated cells, with the ability to self-renew and proliferate for an extended period, and they can differentiate into specific cell types (e.g., osteoblasts, adipocytes, chondrocytes, tenocytes, and myocytes) under appropriate conditions [3]. Recent scientific advancement in stem cell biology and success in clinical trials indicate that stem cell-based therapy is one of the most promising therapeutic strategies [4]. Consequently, current research trends have been directed into developing stem cell-based techniques for periodontal regeneration.

Currently, different semibiological and synthetic substances are used for the proliferation and differentiation of stem cells. When used continuously, the recombinant and synthetic cytokines, growth factors, and other proteins may show side effects and toxic effects [5]. Moreover, even some growth factors can cause malignant formation. Due to different origins of the stimulators, immune-rejection may happen in these cells as well. Also, these reagents usually degrade rapidly and need continuous supplement, which makes them unaffordable and less available for common individuals; therefore, their use in therapeutic tissue engineering is limited. Suitable, costless, and safe alternatives which can help stem cells to integrate into the surrounding environment and reconstruct functional tooth-supporting systems are in need to be developed.

Plant is one of the most essential materials and energy source for humans. It forms the basis of sophisticated traditional medicine system. The use of plants forms the origin of modern medicine. According to the World Health Organization, approximately 25% of modern drugs used in the United States have been derived from plants. Such as aspirin (from willow bark), digoxin (from foxglove), quinine (from cinchona bark), and morphine (from the opium poppy) [6]. Nobel Prize in Physiology or Medicine 2015 for artemisinin has brought phototherapies into the spotlight [7]. Today, artemisinin given in combination with other drugs is the most effective malaria treatment, reinforcing the well-known fact that a considerable portion of drugs produced in current clinical practice have been derived from botanical resources [8]. Plant is still an indispensable reservoir of new molecules with potential therapeutic interest. Recent technological advance in modern herbal medicine coupled with achievement in stem cell therapy has captured the attention of scientists. It may lead to a renewed interest in stem cell-based periodontal regeneration.

## 2. Plant Medicine

Plant is an important reservoir of new molecules with potential therapeutic interest. Botanical products consist of toxins, hormones, or molecules which have biological actives that can be useful to humans. Plant medicine is referred to the study of medicine derived from botanical sources. Phytochemicals are a broad range of biologically active compounds which occur naturally in plants which have vital medicinal and nutritional properties. Comparing to conventional semibiological and synthetic stimulators, plant components have also identically shown promise in diseases such as osteoporosis, neurodegenerative disorders, and other tissue degenerative disorders with the aid of human mesenchymal stem cells (hMSCs) [22].

A series of studies reveal that botanical products have a great number of chiral centers and increase steric complexity compared with either synthetic drugs or combinatorial libraries. Their privileged structures were selected by evolutionary pressure to interact with biological target for specific purposes. The plants often consumed by local communities can be proved safe and less toxic by well-practiced knowledge. It has been proved that herbal remedies show better tolerated adverse effect than synthetic compounds [23]. Since plants are easily available and can be put into industrialization planting at a large scale, the production costs will probably be lower than recombinant growth factors. According to the World Health Organization, up to 80% of the population in developing countries depends on the use of traditional medicine and medicinal herbs for primary health care (WHO, 2007, 2013). Strategic use of herbal remedies as stimulators for proliferation and differentiation in stem cell therapy can bring about cost-effective, highly available, nontoxic alternatives for therapeutic application. It will no doubt lead to a new insight into regenerating periodontal tissues based on the research of stem cells.

**Figure 1:**
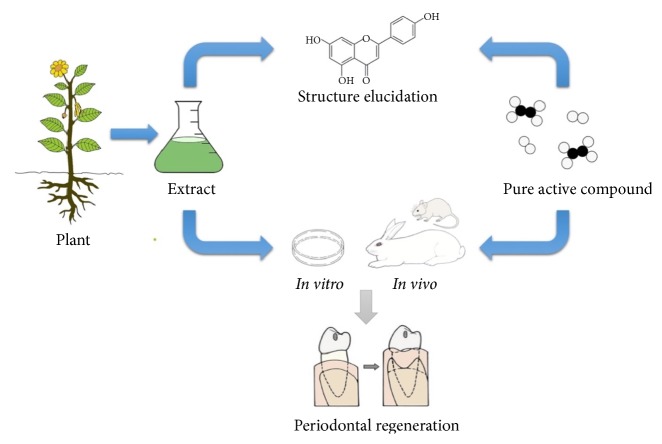
**The procedure of investigating plant medicine for periodontal regeneration using hyphenated techniques**. The plant parts including roots, stems, leaves, and fruits are used in preparations. The process of a plant product therapeutic investigation is summarized as follows: preparation of crude extracts, fractionation for localized and targeted isolation, further purification, and chemical screening using modern techniques such as HPLC, UHPLC, HPLC-MS, bioassays in vitro and in vivo, and clinical evaluation.

Stem cells can be treated with either crude plant extract or pure bioactive compounds. Comparing to directly isolated plant products, it is more possible for pure phytochemicals to serve as leading compound for periodontal regeneration, because the dosage can be more easily qualified. It is argued that, once standardized, the cost of plant extracts prepared from single herbs and herbal mixtures would decrease when put into commercial-scale production. Plant product therapeutic investigation needs to go through a series of procedures (Figure 1).

## 3. Stem Cells Candidates for Periodontal Regeneration

Stem cells can be divided into three categories, embryonic stem cells, somatic stem cells, and induced pluripotent stem cells (iPSCs). Due to legal and serious ethical concerns raised by embryonic stem cells [24], the less predictability of iPSCs' behaviour as well as their tumorigenic properties [25, 26] and somatic stem cells, also known as adult stem cells, have a wider application prospect.

Mesenchymal stem cells (MSCs) are one of the most highly studied adult stem cells. A great number of studies have demonstrated that human MSCs (hMSCs) could avoid allorecognition [27], and their hypoimmunity makes them suitable for allogenic transplantation [28]. According to their tissue of origin, hMSCs for periodontal regeneration can be generally divided into two categories: dental stem cells, e.g., bone marrow-derived mesenchymal stem cells (BMSCs) and non-dental stem cells mainly including periodontal ligament stem cells (PDLSCs) [29], dental pulp stem cells (DPSCs) [30], stem cells from exfoliated deciduous teeth (SHEDs) [31], stem cells from apical papilla (SCAPs) [32], and dental follicle precursor cells (DFPCs) [32].

Cells derived from dental tissues exhibit features similar to those non-dental-derived mesenchymal stem cells [29–31]; however, unlike BMSCs from the bone tissue which undergo continuous remodeling, the cellular potency of progenitor cells derived from dental tissue is more constrained or restricted. Additionally, it is postulated that dental-derived mesenchymal stem cells share similar characteristics with neural crest cells because dental mesenchyme's early interaction with the neural crest [33]. It has been reported that there is no significant difference between the BMSCs and PDLSCs when comparing the alveolar bone regeneration after implantation [34]. However, some studies have demonstrated that, among the PDLSCs, DPSCs, and DFPCs, PDLSCs have the greatest regenerative capacity [35, 36]. Based on a series of landmark studies by Friedenstein and Owen [37–43], PDLSCs are now being regarded as a more suitable cell type for periodontal regeneration. Although PDLSCs exhibit good perspectives for periodontal regeneration, isolating the cells is required at the cost of tooth extraction, which limits their wide range of application in clinic. Clinical application will not only depend on their efficiency and quality of regeneration, but also on their ease of use, accessibility, and cost.

Precursor cells residing in different tissues will differentiate into lineage-specific cells generating various stromal tissues [44]. Moreover, it has also been demonstrated that the adult stem cells have the capacity to differentiate into other cellular lineages beyond their tissues of origin [45, 46]. Each kind of the stem cells has its own superiorities and limitations; using a multitype stem cell approach may reveal beneficial actions. In an ambitious study, stem cells from root apical papilla (SCAP) together with PDLSCs in HA/TCP and “Gelfoam” (absorbable gelatine) carriers were implanted into the cleaned extraction sockets, and, four weeks later, functional root/periodontal complexes were formed which were capable of supporting porcelain crowns in miniature pigs [32].

Screening for effects of phytochemicals on all kinds of hMSCs is extensively studied.

## 4. Plants and Their Bioactive Constituents on hMSCs' Proliferation and Osteogenic Differentiation

Nevertheless, to achieve effective stem cell-based periodontal regeneration strategies, choosing appropriate population of multipotential progenitor cells is far beyond enough. The signaling molecules/inductive morphogenic signals and an appropriate delivery system are also quite essential components [47–49]. As mentioned before, herb extracts, which are used as stimulating factors in periodontal regeneration, have superiorities in price, safety, and availability. A plethora of studies on MSCs carried out to date provide strong evidence to support that plant extracts may have important use in stem cell-based periodontal regeneration.

### 4.1. Proliferation

Plant derivatives, despite their natural origins, could demonstrate cytotoxicity, suppressing cell proliferation even inducing apoptosis. To study the effects of plant-derived extracts or chemicals, evaluating the cytotoxicity and exploring the optimal concentration are crucial for achieving the desired therapeutic goal. Effects may vary under same concentration due to cell type, inoculation number, and culture conditions [50]. Some plant-derived substances exhibit strong osteogenic capacity. However, they inhibit cell proliferation, posing a major limitation for their application. It has been reported that, at concentration of 10^−8^ - 10^−5^ M, Resveratrol promoted cell proliferation and osteoblastic differentiation, while, on high concentration (10^−4^ M), it exerted a strong suppressing effect [51]. This effect may be related to direct cytotoxicity and it came often. However, salvianolic acid B (Sal B), extracted form* Salvia miltiorrhiza*, was observed not affecting the viability of hMSCs over a wide range of concentrations, so it can be inferred that Sal B had no cytotoxicity to hMSCs [52]. Another study investigating flavonoids of* Herba epimedii* (HEF) showed that, with the increase of concentration, HEF moderately inhibited the proliferation of MSCs, while opposite trend occurred regarding their osteogenesis [53]. In another study, Li, Zhang et al. found that the ethanol extracts of the fruit of* Ligustrum lucidum* inhibited the proliferation of human bone marrow-derived mesenchymal stem cells (hBMSCs) in a dose-dependent way and presented a cytotoxic effect at a concentration of ≥200 *μ*g/mL [54].

### 4.2. Osteogenic Differentiation

Costa, Amorim et al. conducted a systematic review on effects of plants on osteogenic differentiation and mineralization of periodontal ligament cells [55]. Six different types of plants were reported in this study, namely,* Morinda citrifolia, Aloe vera, Fructus cnidii, Zanthoxylum schinifolium, Centella asiatica, *and* Epimedium* species. Gene or protein expression of RUNX2 and COL1 was upregulated in four and five studies, respectively. Other genes responsible for bone metabolism were also evaluated, such as ALP, OCN, OSX, OPN, RANKL, and OPG. In addition, five studies observed the mineral deposition.

There are other articles which reported increased expression of bone markers and mineral deposition on mesenchymal stem populations from human, mainly based on BMSCs and also DPSCs [56–60]. Ferutinin, a phytochemical found in plants belonging to the* Ferula* genus, particularly in the roots of* Ferula* hermonis, significantly increased protein level expression of OPN, OCN, and COL1 after 14 days culture on DPSCs in comparison with the control group. And the result also displayed an increase of calcium deposition [60]. Another study investigated total flavonoids of* Herba epimedii*, a traditional Chinese herbal medicine, commonly used for treatment of osteoporosis in China, promoting hBMSCs osteogenesis via enhancing bone marker gene expression such as OPN and OCN [58]. Naringin, the main effective component of* Rhizoma drynariae*, resulted in inducing hBMSCs osteogenic differentiation through increasing the expression of these osteogenic differentiation markers (ALP, COL1, OPN, and OCN). And the ALP and Von Kossa staining assays revealed it increased the quality and quantity of calcium node formation [56].

## 5. Functions and Action Mechanisms Involved

The signaling pathways for functions of plant-derived substances on hMSCs has been a subject of increasing interest, such as BMPs signal pathways, MAPK signal pathways, and Wnt/*β*-catenin pathways, and there are various connections between the signaling pathways.

### 5.1. Activation of MAPK Signaling Pathways

The activation of JNK-ERK-p38 MAPK pathway is known to play a crucial role in osteogenesis. The involvement of MAPK signaling pathway is shown in different hMSCs treated with plant extracts or phytochemicals. Sal B, as mentioned above, was used in Traditional Chinese Medicine (TCM) for cardiovascular diseases. Xu, Xu et al. discovered that it stimulated osteogenesis of hMSCs through activating ERK signaling pathway [52]. ERK1/2 is constantly activated during osteogenic differentiation. Additionally, ERK is considered to regulate osteoblast proliferation, differentiation, and apoptosis by regulating expression of cell cycle regulators as well as activity of Runx2. It has been reported that the skeletal-specific transcription factor Runx2 is a target of ERK1/2 pathway [61–63]. In this study, Sal B elevated osteogenic ability of hMSCs via inducing the phosphorylation of ERK1/2, which successively upregulated the expression of transcription factors Runx2.

Activation of the JNK pathway is correlated to apoptosis and cell death [64–66]. There is evidence that osteoblast differentiation was stimulated by activation of ERK and JNK [67]. Fucoidan, a vegetal sulfated polysaccharide extracted from brown seaweed, was reported to promote osteoblast differentiation in human alveolar bone marrow stem cells by activation of JNKs and ERKs. Moreover, the regulation of the JNK and ERK pathway does not appear to be an isolated cascade. The effect was associated with increasing phosphorylation of Smad 1/5/8 and BMP2 mRNA expression. Because in this study, JNK and ERK inhibitors diminished fucoidan-induced Smad 1/5/8 phosphorylation and BMP2 mRNA expression [68]. It is known that the p38 MAPK pathway is essential in controlling osteoblast differentiation and skeletogenesis [69]. Amentoflavone, a bioflavonoid found in a variety of traditional Chinese medicines such as Gingko and* Selaginella tamariscina*, enhanced the proliferation, alkaline phosphatase activity, and mineralization of hMSCs via JNK and p38 MAPK pathway [70].

### 5.2. Activation of BMP and Wnt/*β*-Catenin Pathways

It is known that BMPs plays a vital role in bone formation signaling pathways [71]. Myricetin, a flavonoid compound, was proved to induce osteogenic differentiation of hBMSCs by activation of the Wnt/*β*-catenin pathway and increasing the expression of several downstream genes including T-cell factor-1 (TCF-1) and lymphoid enhancer factor-1 (LEF-1) [72].

Naringin, as shown previously, exerted osteogenic effects on hBMSCs [56]. Another study is carried out on rat osteoblastic UMR-106 cells, suggesting that stimulation of Wnt/*β*-Catenin signaling, which leads to the activation of lymphoid enhancer factor (LEF)/T-cell factor (TCF) transcription factors, which were plausible mechanisms for bone generation [73].

As the study afore mentioned, HEF was shown to upregulate osteogenesis of hMSCs [53]. And there is evidence that Ginkgo biloba extracts (GBE), from traditional herbal medicine, were capable of promoting proliferation and osteogenic differentiation of hBMSCs [74]. Osteogenic effects of the two studies were all linked to upregulating the transcriptional level of bone morphogenetic protein 4, RUNX2, *β*-catenin, and Cyclin D1, which indicates that that BMP and Wnt/*β*-catenin signaling pathways were involved in the cascades. Moreover, both of the studies carried out the loss-of-function assay, which found that the osteogenic effect was inhibited by the inhibitor of BMP and Wnt/*β*-catenin signaling pathways Noggin and DKK-1, respectively. Both of the studies suggest involvement of BMP and Wnt/*β*-catenin pathways.

**Figure 2:**
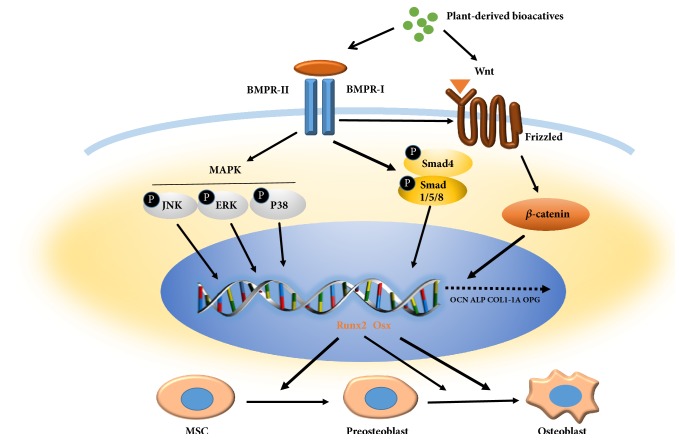
**Schematic diagram for the activation of BMPs, Wnt, and MAPK pathways by phytochemicals treatment**. Multiple signaling pathways regulate human mesenchymal stem cells osteoblastic differentiation, mainly including BMPs, Wnt, and MAPK. Some plant-derived bioactives could activate certain signaling pathway. Through the coordination of other pathways, then downstream molecules translocate into the nuclei where there is interaction with transcription factors such as Runx2 and Osx via which they regulate bone-related genes expression and play different roles at different stages of osteogenesis.

The canonical Wnt/ *β*-catenin signaling and BMP signaling are key pathways for regulating bone formation and remodeling [75–77]. The process of osteogenesis can be divided into three major stages in brief, the osteoprogenitor stage, the preosteoblast stage, and the mature osteoblast stage [78]. The Wnt/*β*-catenin signaling is related to the early stage of osteogenesis and the further differentiation of mature osteoblasts and calcium deposition [79]. In osteogenesis, the BMP signaling drives osteoblast progenitor cells into mature osteoblasts [80, 81] and further differentiation by increasing expression of ALP [82, 83] and calcium mineralization [84]. Also, BMP was known to be involved in Wnt/ *β*-catenin signaling pathways [85]. As a matter of fact, BMP-4 induces the expression of Runx2, the downstream regulator of the BMP pathway [86–88], which then regulates the expression of OSX in osteoblastic differentiation [85, 89, 90]. It has been found that Runx2 regulate osteogenic differentiation of MSCs via integrating Wnt/*β*-catenin [91, 92]. Therefore, Runx2 may act as a cross-talking regulator between the BMP and Wnt/*β*-catenin signaling pathways. As described above, plausible underlying mechanisms of action could be investigated from the three main signaling pathways (Figure 2).

## 6. Plant Derivatives in Tissue Engineering

Following the idea “from bench to bedside and back”, there has been increasing interest in translating in vitro basic research investment into in vivo reward [93, 94]. Although many studies have proved that plant medicine may potentially revolutionize regenerative medicine and tissue engineering, there is still a gap between basic study and clinical research. The clinical use of stem cells and growth factors is often hindered by delivery problems. A satisfactory delivery system must be developed before clinical application. Nevertheless, effects of such delivery system on stem cells are scantily reported. Plenty of research on relevant cell lines and in vivo studies concerned provide the basis for further studies directly on stem cells.

### 6.1. Biomaterials-Based Approach

#### 6.1.1. Plant Derivatives as Osteoinductive Bioactive Factors

Scaffolds are extensively studied for delivery of growth factors and stem cells. It can be combined with living tissue cells and maximize the beneficial effects of different stimulating factors when implanted into organisms. Scaffolds can provide necessary space for cells to grow on its porous structure and mechanically support the cell adhesion, proliferation, location, and differentiation. Moreover, as supportive carriers, scaffolds can also conduct sustained release of bioactive factors [95]. The scaffold should be biocompatible and degrade at a desired rate to support the periodontal defects until fully achieving regeneration [96]. The triangle mode of taking scaffold material as framework, compounding stem cell, and assisting with growth factor in combination with plant extracts will bring forth a brand new idea in tissue engineering [97].

There have been various examples of plant-derived substances incorporating to scaffolds, which proves they could effectively improve the bioactivity of different biomaterials as well as regulating protein or gene expression responsible for osteogenic differentiation. These phytochemicals could work as growth factors, reducing the need for expensive and difficult biological moieties. The most frequently used scaffold materials are hydroxyapatite or the complex with other components, synthetic polymers (e.g., sulphonated poly aryl ether ketone and polylactide glycolic), or the polysaccharides (e.g., chitosan). Although they all proved to be highly biocompatible, they show no or little effectiveness of bone induction. The plant derivatives incorporating to these materials would make up for their poor osteoinductive abilities. Even some studies indicate the structure of the scaffold will be changed when incorporated with plant-derived bioactives.

The responses of Gu-Sui-Bu (*Drynaria fortunei* J. Sm) immobilized modified calcium hydrogen phosphate (GI-MCHP) on bone cells have been evaluated in an* in vitro* study [98]. Gu-Sui-Bu is a kind of TCM in treatment of bone disorders. In this study, Gu-Sui-Bu still preserved its beneficial effects on osteoblasts, which suggests that GI-MCHP may be a satisfactory delivery system for Gu-Sui-Bu.

The extracts of* Cissus quadrangularis* (CQ) and* Butea monosperma* (BM) incorporated with sulphonated poly aryl ether ketone (SPAEK) sponges is reported to own good biocompatibility. In this study, both SPEAK-CQ and SPEAK-BM remarkably increased the ALP activity, which is an early marker of osteogenic differentiation and mineral deposition [9]. This result incites that the scaffolds with phytochemicals can be applied in cases like regenerating resorbed dental alveolar ridge, to promoting periodontal regeneration.

M.* tenuiflora cortex*, a kind of plant easily harvested in Mexico, has been used for decades as a remedy in the treatment of wounds and burns of the skin. In a study, different proportions of M. tenuiflora were incorporated into chitosan and divided into three groups [10]. Incorporating M.* tenuiflora cortex* to chitosan would obtain a composite containing the beneficial properties of the two components. It is interesting to note that 80/20 chitosan/M.* tenuiflora* composite had the best performance compared with the other two groups, as this kind of composite has more homogenous porosity and the result indicates that the two components involve a synergistic effect on cell viability and proliferation. So it can be speculated that the proportion of plant bioactives are added may not only affect the osteogenic capacity, but make a difference to the structure of the scaffolds, which leads to changes in biological functions.

There have been various published studies regarding scaffolds combined with botanical substances contributing to periodontal regeneration. To move beyond observations of effects in in vitro fabricated delivery systems, researchers have established animal models to evaluate the efficiency and further biocompatibility. Xu, Guo et al. incorporated Astragalus polysaccharides with chitosan or polylactic acid to deliver BMSCs to horizontal periodontal defects in dogs. It was observed that not only did the two novel fabricated scaffolds induce osteogenesis* in vitro*, but also the amount of new bone formation and the rate of new bone filling to the defect height increased [99].

Safflower seed extracts have been evaluated to treat intrabony defects. Kim, Kim et al. evaluated the effect of safflower seed extracts (SSE) added to collagen sponges (SSE/Col) on the regeneration of periodontal tissue of preclinical 1-wall intrabony defects model in beagle dogs [11]. They compared the SSE/Col group with phosphate-buffered saline/collagen (buffer control) and root planning only (surgical control). Eight weeks after surgery, which is a sufficient healing period for healing bone regeneration, the histologic and histometric analyses were performed. Although SSE/Col group was not statistically different form buffer control in alveolar bone formation, it is noticeable that the percentage of intrabony cementum was more than the other two group, which is the true cementum with perpendicularly inserted fibers. The result shows that the SSE/Col structure had periodontal regeneration potential. More recently, the same research group studied the effect of polylactide glycolic acid nonwoven membrane containing safflower seed extracts (SSE/PLGA) using the same grouping and evaluation method [12]. The results were similar; however no significant difference was observed between the SSE/Col and buffer control. Compared with surgical control group, the SSE/PLGA could relatively promote the regeneration of alveolar bone and cementum in intrabony periodontal defect.

Icariin, a crenelated flavonol glycoside contained in the herb of the genus* Epimedium*, has proved a potent stimulator for osteogenesis. Bi, Fan et al. investigated the icariin-loaded chitosan/nano-sized hydroxyapatite system on New Zealand rabbits. It was found that icariin and CS/HA composite did not affect each other's performance, and the system promoted osteogenesis on BMSCs by increasing ALP activity and mineralized nodules formation [13].

In a recent pilot clinical trial, the efficacy of a composite graft composed of bovine-derived hydroxyapatite (HA) and* Cissus quadrangularis* was evaluated. The author compared the graft material (experimental group) to HA alone (control group), after scaling and root planning. Though both groups exerted beneficial effects, the experimental group only showed a slightly better performance [14]. The potential of system still needs future studies to clarify, because the small sample size (n = 20) used in this study may give rise to result error. The plant derivatives work as osteoinductive bioactive factors that contribute to application in tissue engineering are summarized in Table 1.

#### 6.1.2. Plants Derivatives as Scaffolds

Another condition is that the scaffold itself is made from plant. Plant extracts can not only be utilized as stimulating factors to enhance the cells attachment, proliferation, and differentiation, but also act as vital components of the scaffold materials. Biomaterials used today from natural origin have self-assembly and cross-linking properties. They also have an innate ability to mediate scaffold degradation and interact with cells [95]. Various studies suggest that it could be a feasible and meritorious strategy for periodontal regeneration. Most plant-derived polymers such as cellulose, alginate, and agarose have typically been referred to as “hydrogels”, as they are composed of a polymeric network which could contain up to 99 per cent or higher water content. Due to their highly hydrophilic nature, as a result, their swelling capability in water allows them to provide an environment that mimics the highly hydrated natural extracellular matrix (ECM) [100], making them potential therapeutic agents for various tissue engineering applications.

Cellulose is one of the most abundant renewable materials, which is widely distributed in higher plants. Recent studies have shown that cellulose scaffolds are biocompatible and show promising potential as a biomaterial for scaffold. Various forms of cellulose have been shown to have utility in both in vitro and in vivo studies [101–105]. As well, the scaffold itself was able to retain much of its original shape and structure over the 8-week study [106]. Importantly, the study also shows the scaffold clearly has a proangiogenic effect, promoting the growth of functional blood vessels throughout the implanted biomaterial, which provides a favorable opportunity for bone regeneration.

Agarose, a polysaccharide with thermosensitive characteristic, has been used to engineer an injectable hydrogel for the formation of cartilage [15]. The study compared the alginate, agarose, and collagen gel for bone morphogenetic protein 2 (BMP-2) genetic tissue engineering* in vivo*. Although the three groups all promoted osteogenesis, it is noticeable that the agarose-gene-transduced BMSC gel was found to contain much more hyaline cartilage.

Some of the plant-derived biomaterials could not only contribute to bone formation through their osteoconductive properties, they could also directly intervene in the bone repair mechanisms, and at the same time serve as growth factors for osteoinduction, for example, acemannan sponges and soybean granules.

Acemannan is a major carbohydrate fraction of Aloe vera gel. The effects of acemannan both used as a bioactive molecule or a scaffold for periodontal regeneration has been investigated, respectively, in vitro and in vivo. In vitro study, primary periodontal ligament cells treated with acemannan showed upregulation of osteogenic-related genes expression. In vivo study where premolar class II furcation defects were made in four mongrels, new alveolar bone, cementum, and periodontal ligament formation were significantly accelerated by acemannan sponges [16].

Another new class plant-derived biomaterial based on soybeans has shown intrinsic osteogenic bioactivity and anti-inflammatory effects in vivo trails [17]. It also possessed controlled release of contained isoflavone and favorable biodegradation rate. Merolli, Nicolais et al. have done a research on soybean granules implanted in rabbit femoral epiphyseal defects. Over 8 weeks, new trabecular bone regenerated with features distinct from sham-operated group (control group). The result indicated them of great bone regeneration potential. Coupled with its relatively low cost and easy preparation procedures, it may become a potent active osteoinductive material for periodontal regeneration.

In addition to single-component scaffolds, composites of diverse plant-derived polymers or composites of various plant-derived polymers and other synthetic biomaterials are also widely studied.

Composites of diverse plant-derived polymers could show improved performance and balance the strengths and weaknesses of individual components. Ko, Sfeir et al. synthesized single-component scaffolds of plain cellulose, plain chitosan, and plain alginate as well as composite scaffolds of cellulose–alginate, cellulose–agarose, cellulose–chitosan, chitosan–alginate, and chitosan–agarose via lyophilization technique to assess their suitability as tissue engineering scaffolds. Cross-sectional SEM shows that the pore size and pore size distribution within different composite scaffolds varied. Although cell morphology varied on different materials, it is shown that HeLa cells attached and proliferated well on the scaffolds [107].

A growing body of literature suggests that composite scaffolds comprising plant-derived components and other kind of non-plant-derived biomaterials, such as chitosan and bioceramics, may confer substantial benefits to tissue engineering. A novel delivery system fabricated by incorporating* Cissus quadrangularis* (CQ) extracts with alginate (Alg) and O-carboxymethyl chitosan (O-CMC) (Alg/O-CMC/CQ-E scaffold) has been studied on hMSCs in vitro. Compared with the scaffolds without plant extracts, the Alg/O-CMC/CQ-E group significantly enhanced proliferation and attachment of initial cells. Moreover, even without osteogenic media supplement, the hybrid scaffold possessed an osteoinductive property [18]. The results show that the Alg/O-CMC/CQ-E scaffold possesses excellent osteoinductive ability and desirable physicochemical property, which makes it a promising candidate for bone tissue engineering.

Genipin, extracted from the fruit of* Gardenia jasminoides Ellis*, is considered as favorable cross-linking agent. It could cross-link compounds with primary amine groups such as proteins, chitosan, and collagen. Genipin-crosslinked chitosan (GEN-chitosan) hydrogel is also a promising delivery system for tissue regeneration. Different genipin concentrations did not change the mechanical properties of the cross-linked chitosan scaffold structures as well as morphology and porosity, but the degradation profiles [19]. In addition, another study demonstrated that optional addition of nano hydroxyapatite to the formulations could significantly enhance their osseointegrative capacity both in vitro and in vivo [20].

Alginate is a kind of polysaccharide mainly derived from brown algae, certain seaweeds, which has been used in various biomedical applications, including cells or growth factor delivery. Srinivasan, Jayasree et al. successfully fabricated alginate/nano bioactive glass ceramic particle (nBGC) composite scaffolds using lyophilization technique [21]. Osteosarcoma (MG-63) and human periodontal ligament fibroblast cells (hPDLFs) exhibited better cell attachment in virtue of the incorporation of nBGC when seeded onto the scaffolds. The hPDLFs also showed distinct osteoblast-like behaviour with enhanced alkaline phosphatase activity in comparison to the control alginate scaffolds, suggesting the composite scaffold can act as a suitable bioactive matrix for periodontal regeneration. The plant derivatives serve as scaffolds that contribute to application in tissue engineering are summarized in Table 2.

### 6.2. Biomaterials-Free Approach

In contrast with relying on biomaterials to achieve periodontal regeneration, the novel tissue engineering methodology called cell sheet also has yielded success in regenerative medicine [108–111]. Cell sheet constructs functional tissue without any artificial scaffolds. The technique avoids damaging critical cell surface proteins such as ion channels, growth factor receptors, and cell-to-cell junction proteins [112]. Remarkably, the first human experiment using PDLSCs was performed based on cell sheet delivery [113]. It has been reported that Osthole, a coumarin-like derivative extracted from Chinese herbs, promoted osteogenic properties and induced the formation of cell sheets in vitro using human periodontal ligament stem cells (hPDLSCs) and jaw bone marrow mesenchymal stem cells (JBMMSCs) at a certain concentration. In vivo study, Osthole-stimulated and unstimulated cell sheets were subcutaneously transplanted into the dorsal region of nude mice, and the result showed that Osthole-mediated JBMMSC and PDLSC sheets formed more new bone than those obtained without Osthole [59]. Plant-derived compounds utilized in the field of cell sheet remain largely unexplored; further more research is required to investigate its true potential.

## 7. Estrogenic Effects of Plants on Stem Cell-Based Periodontal Regeneration

Estrogen plays a crucial role in the maintenance of skeletal homeostasis for both man and women. Its deficiency is considered as one of the major causes of postmenopausal osteoporosis. Osteoporosis is believed to be one of the risk factors for periodontal disease and tooth loss [114, 115], especially postmenopausal osteoporosis [116, 117]. As a result, it has been hypothesized that estrogen deficiency has some links with periodontal diseases [118]. It has been demonstrated that estrogen deficiency will cause impaired osteogenic differentiation of PDLSCs [119]. In addition, estrogen deficiency could result in inflammation and decreased synthesis of fibroblasts which produce collagen [120]. Also, a study has shown estrogen enhanced the bone regeneration potential of PDLSCs derived from ovariectomized osteoporotic rats (estrogen deficiency animal model) [121]. Another study has discovered that estrogen could stimulate the periodontal ligament cells osteogenesis by increasing ALP activity, osteocalcin distribution, and the formation of mineralized nodules [122]. Furthermore, it has been reported that both ER*α* and ER*β*, which are estrogen receptors, were involved in the process of osteogenic differentiation of PDLSCs [123]. As a result, estrogen therapy may help estrogen deficiency impaired periodontal tissue get regeneration [124].

The estrogen we usually use for clinical Hormone Replacement Therapy (HRT) is mainly extracted from the urine of pregnant mare. Unfortunately, there comes the problems of side-effect, such as increased risk of breast cancer and endometrium hemorrhage [125, 126]. Phytoestrogen is a class of aromatic sterols distributed in plants widely. Their metabolites and themselves can bind to estrogen receptors in mammals and play a similar role of the endogenous estrogen. Considering the negative side effects, phytoestrogen has been given further attention as a potent alternative to estrogen replacement therapy. Accordingly, it has a promising prospect in regenerating periodontal tissue damage due to estrogen deficiency. A host of studies have exhibited that phytoestrogens exerted effects on bone regeneration [127–130], but few cases have been reported concerning their effects on periodontal diseases. It has been reported that Tofu liquid waste as a natural phytoestrogen could reduce the risk of periodontitis. The study reported that the dosage of different Tofu liquid waste could reduce the number of osteoclasts and the width of periodontal ligament space and increase the number of osteocytes and the alveolar bone height in ovariectomized rats [131]. More cases are needed to prove phytoestrogen of practical value in periodontal regeneration.

*Challenges and Prospects*. Not only intrinsic (genetic) but also extrinsic factors affect the plant products. Using different solvents and methods to prepare phytochemicals may produce different outcomes and even can lead to adverse effects. Environmental and cultural factors as well as the process of preparation together influence the quality and the quantity of bioactive constituents, which may lead to undesirable side effects and low bioactivities [22]. Clinical applications are also challenging due to the variability and complexity of bioactive constituents present in herbal formulations. Accordingly, functional components in the botanical products need to be clarified. Another condition may be the plant medicine with rough processed mixtures of different ingredients, and in actual manufacturing practice they need to be standardized under highly regulated. Standardization is the principle for putting plant-derived chemicals into clinical use to ensure quality, efficacy, safety, and reproducibility.

Acellular cementum is a unique tissue, while cellular cementum and bone share some similarities, although there are still morphological, functional, and biochemical differences between the two tissues [132]. It is controversial when a kind of phytochemical promotes osteogenesis, it can also promote cementogenesis. Wnt signaling has been implicated in increasing osteogenesis by controlling mesenchymal stem cell or osteoblastic cell functions. It has been reported that activation of the canonical Wnt signaling pathway inhibits cementum regeneration [133]. In terms of how the activators inhibit cementum regeneration, the mechanism may be because enhancing DDK1 expression in an Osterix-dependent way [134] or via regulation of expression of Runx2 [133]. However, another study has shown that activation of the Canonical Wnt signaling pathway can induce cementum regeneration* in vivo* and* in vitro* cementogenic differentiation of hPDLCs [135]. There are two markers that have been found to be specifically expressed in cementogenic cells: cementum attachment protein (CAP) [136] and cementum protein 1 (CEMP1; also called CP23) [137]. Both markers have been identified in progenitor cells of the PDL as well as in cementoblasts [138, 139]. The expression of cementum-associated genes should also be examined when cells treated with plant-derived constituents.

As periodontium is a highly vascularized tissue, the development of microvasculature and microcirculation is crucial for bone regeneration. A study systematically reviewed several herbal medicine:* Salvia miltiorrhiza Bunge*,* Angelica sinensis, Astragalus membranaceus Bunge, and Puerarin radix* [140]. They all have positive effects on bone formation, and the possible mechanisms may be related to their ability to promote angiogenesis via an effect on substances such as VEGF. Many reports over the years have demonstrated a close link between angiogenesis and bone growth. Consequently, it can be speculated that herbal medicine with angiogenic properties may be similarly beneficial for bone formation and periodontal regeneration, so plant-derived constituents reported beneficial for angiogenesis deserve more investigation in follow-up studies.

## Figures and Tables

**Table 1:** Plant derivatives work as growth factors for application in tissue engineering.

**References**	**Plant-derived substances**	**Delivery system**	**Study design**	**Cell line/Animal model**	**Conclusion**
[9]	*Cissus quadrangularis* and *Butea monosperma *	sulphonated poly aryl ether ketone (SPAEK) sponges	*in vitro*	SaO2 (Human Osteosarcoma cells)	The system exhibited good biocompatibility towards the cells, and the sponges successfully delivered the phytochemicals, which can work as growth factors.

[10]	M. *tenuiflora cortex*	chitosan	*in vitro*	rat primary osteoblasts cavaria cells	The combining the chitosan and M. *tenuiflora cortex* in scaffolds has potential for bone tissue regeneration. The M.* tenuiflora cortex* promoted the proliferation and differentiation of osteoblasts cells.

[11]	Safflower seed extracts	collagen	*in vivo*	beagle dogs	Safflower seed extracts may contribute to bone formation and appears to have potential for stimulating periodontal regeneration including new cementum.

[12]	Safflower seed extracts	polylactide glycolic acid non-woven membrane	*in vivo*	beagle dogs	Surgical application of PLGA non-woven membrane with or without safflower seed extracts could promote the regeneration of alveolar bone and cementum in intrabony periodontal defects.

[13]	Icariin	chitosan/nano-sized hydroxyapatite (IC-CS/HA)	*in vitro/in vivo*	hBMSCs/New Zealand rabbits	Icariin-CS/HA is believed to be an optical bone repair scaffold for tissue engineering.

[14]	*Cissus quadrangularis*	bovine-derived hydroxyapatite	*in vivo*	humans	The potential of the system still remains to be researched.

**Table 2:** Plant derivatives work as scaffolds for application in tissue engineering.

**References**	**Plant-derived substances**	**Delivery system**	**Study design**	**Cell line/Animal model**	**Conclusion**
[15]	Agarose	agarose gel mixed with adenovirus-mediated human BMP-2 gene transduced bone marrow stromal cells	*in vivo*	athymic mice	The agarose-gene-transduced BMSC gel was found to contain much more hyaline cartilage than the alginate and collagen gel.

[16]	Aloe vera gel extracts	acemannan sponges	*in vivo*	mongrels	Acemannan could be a candidate osteoinductive biomaterial for periodontal tissue regeneration.

[17]	Soybean	soybean granules	*in vivo*	New Zealand rabbits	Soybean-based biomaterial may become a potent active osteoinductive material for periodontal regeneration.

[18]	Cissus quadrangularis (CQ) extracts with alginate (Alg)	alginate/O -carboxymethyl chitosan/ *Cissus quadrangularis *scaffold	*in vitro*	hMSCs	The hybrid scaffold owned a substantially osteoinductive capacity, which could serve as a potential candidate for bone tissue engineering therapeutics.

[19]	Genipin	genipin-crosslinked chitosan hydrogels	*in vitro*	osteosarcoma (MG-63) cells, hMSCs	Genipin cross-linked chitosan scaffolds are suitable systems for bone tissue engineering. Different genipin concentrations effectively change the degradation profile, the structural and mechanical properties of the scaffolds.

[20]	Genipin	electrospun from chitosan crosslinked with genipin	*in vitro/in vivo*	murine mesenchymal stem cells/CD1 female mice	The presence of HA in the CTS-GP scaffold significantly enhanced their osseointegrative capacity, making it unique biomaterial for repair of bone defect.

[21]	Alginate	alginate/nano bioactive glass ceramic composite	*in vitro*	MG-63 cells, human periodontal ligament fibroblasts	The results suggest that these biocompatible composite scaffolds have possible relevance for periodontal tissue regeneration.

## Abbreviations

ALP: Alkaline phosphatase
AMPK: Adenosine 5'-monophosphate-activated protein kinase
BMPs: Bone morphogenetic protein
DDK-1: Dickkopf-1
ERK: Extracellular regulated protein kinases
JNK: c-Jun N-terminal kinase
HPLC: High-performance liquid chromatography
UHPLC: Ultra-high-performance liquid chromatography
OCN: Osteocalcin
OPG: Osteoprotegerin
OPN: Osteopontin
OSX: Osterix
RANKL: Receptor Activator for Nuclear Factor-*κ* B Ligand
UHPLC: Ultra-high-performance liquid chromatography
VEGF: Vascular endothelial growth factor.
